# Green talent management as a driver of public sector well-being: insights from civil servants’ job satisfaction in China

**DOI:** 10.3389/fpsyg.2025.1624891

**Published:** 2025-12-24

**Authors:** Mi Zhou, Biying Wang, Rumeng Zhang

**Affiliations:** 1 School of Public Administration, Lanzhou University of Finance and Economics, Lanzhou, China; 2 School of Business Administration, Jiangxi University of Finance and Economics, Nanchang, China

**Keywords:** green talent management, green organizational identity, artificial intelligence, public sector, civil servant, job satisfaction and public administration

## Abstract

**Introduction:**

Enhancing public service quality and societal well-being increasingly relies on the strategic development of human capital within the public sector. Although green talent management (GTM) has been widely applied to promote sustainability and performance in for-profit organizations, its mechanisms and effects in public administration remain insufficiently examined. This study investigates how GTM influences civil servants’ job satisfaction, highlighting the roles of green organizational identity and artificial intelligence (AI).

**Methods:**

Data were collected from 347 civil servants in China’s public administration sector using a structured survey. Hierarchical regression analyses were conducted to test the mediating effect of green organizational identity and the moderating effect of AI.

**Results:**

Both green hard talent management and green soft talent management significantly enhanced civil servants’ job satisfaction. Green organizational identity mediated these relationships. In addition, AI moderated the effect of green hard talent management on green organizational identity.

**Discussion:**

The findings offer empirical evidence that GTM contributes to improving job satisfaction by strengthening organizational identity among public sector employees. They further suggest that AI can amplify certain GTM effects, providing deeper insight into how sustainability-oriented talent practices can support human capital development and public sector modernization.

## 1 Introduction

As the backbone of society, providing essential services to the public, safeguarding the public interest, and promoting social equity and justice, the work of civil servants is vital to the development and stability of a country or region (Blijleven and Van Hulst, 2021; Neo et al., 2023; Van Der Steen et al., 2018). Improving the satisfaction of civil servants is a critical issue for managers to address, as increased satisfaction among civil servants is often linked to higher levels of public service motivation. Highly satisfied civil servants can more efficiently implement government policies, facilitate the smooth execution of public services and projects, and enhance the effectiveness and efficiency of government governance (Lee and Jin, 2024; Vandenabeele, 2014; Wang and Brower, 2019). Relevant literature also confirms the existence of the satisfaction mirror, indicating that improving civil servant satisfaction can effectively enhance citizen satisfaction (Petrovsky et al., 2023). Moreover, highly satisfied civil servants are more likely to participate in and support administrative reforms and innovations, driving continuous improvement and optimization within public departments, and adapting to social and technological changes (Braams et al., 2024; Cantarelli et al., 2016; Kroll and Vogel, 2014).

Environmental protection and sustainability are significant aspects that public departments need to focus on, as they are closely linked to public health, welfare, and social stability (Lin et al., 2022). In fact, environmental protection is a global issue, involving human health, ecosystem balance, sustainable resource utilization, and the sustainable development of the global economy (Azhar and Yang, 2022; Rangarajan and Rahm, 2011). Recently, green talent management has emerged as a new management concept increasingly adopted by public organizations, which involves strategically enhancing and developing employees’ environmental awareness, capabilities, and skills to achieve organizational environmental goals and promote sustainable development, and the effectiveness of this management concept has been preliminarily explored (Ma et al., 2023; Odugbesan et al., 2023; Ogbeibu et al., 2022; Umair et al., 2023).

However, existing studies on green talent management (GTM) have been conducted almost exclusively in private-sector contexts, leaving its applicability and effectiveness in the public sector largely underexplored. Public organizations operate under distinct institutional logics—such as bureaucratic structures, career-based employment systems, and strong norms of procedural fairness—which may fundamentally alter how civil servants perceive and respond to GTM practices. Therefore, understanding how GTM influences civil servants’ job satisfaction represents a meaningful research gap that has not been addressed in prior studies.

Additionally, based on previous relevant studies, green talent management can be further divided into green hard talent management (GHTM) and green soft talent management (GSTM). The distinction between GHTM and GSTM is grounded in the classic hard–soft HRM framework, which differentiates control-oriented, performance-driven practices from people-oriented, developmental ones. Accordingly, GHTM emphasizes standardized procedures, strict environmental performance evaluations, and hierarchical control, whereas GSTM focuses on employee participation, communication, and support for well-being and capability development (Odugbesan et al., 2023; Ogbeibu et al., 2022). Yet prior literature has not examined whether these two distinct approaches function similarly or differently within public organizations. Clarifying whether GHTM and GSTM produce divergent effects on civil servants’ job satisfaction not only addresses an empirical gap but also contributes theoretically by testing whether GTM’s dual-path framework applies to the public sector.

Overall, this study aims to explore the impact of two green talent management strategies (GHTM and GSTM) on civil servants’ job satisfaction and to construct a moderated mediation model to examine the roles of green organizational identity and artificial intelligence. This study contributes to the existing literature in several ways. First, this study is among the early empirical investigations to examine green talent management within public organizations, particularly focusing on civil servants. Previous studies have provided insights into the implementation effects of green talent management but have been validated in general organizations, lacking empirical evidence from public organizations. By differentiating the effects of GHTM and GSTM on civil servants’ job satisfaction, this study supplements and improves related literature.

Second, this study examines the process mechanisms through which green talent management exerts its effects by drawing on organizational identity theory. According to this theoretical perspective, individuals define themselves partly through their membership in the organization and derive a sense of meaning, belonging, and emotional attachment from this association (Nunes et al., 2024; Ran and Golden, 2011). Green organizational identity is a segmented concept in the context of environmental protection, reflecting employees’ recognition and sense of belonging to the organization’s environmental protection and sustainable development efforts (Chen, 2011; Song et al., 2019). Organizational identity theory suggests that when employees internalize the organization’s mission and perceive alignment between their personal values and the organization’s environmental goals, they experience stronger commitment, greater work meaningfulness, and more positive work attitudes. Consistent with this theoretical logic, we propose that green organizational identity serves as a key mediating mechanism linking green talent management to job satisfaction.

Third, this study also investigates the moderating role of artificial intelligence (AI) in the effectiveness of green talent management. With the development and maturity of technology, governments worldwide have begun to deploy AI technologies to improve e-governance and achieve agile governance in the digital age. AI technologies are expected to assist in managing public affairs, such as smart city management, policy evaluation and decision support, and big data-based public health management (Li et al., 2023; Taeihagh, 2021; Wirtz and Müller, 2019). The application of AI in the public sector may also impact human resource management (Allal-Chérif et al., 2021; Rožman et al., 2022). Existing research suggests that AI may introduce both opportunities and challenges for talent management. On the one hand, AI-enabled tools could improve transparency, standardization, and data-driven decision processes; on the other hand, concerns related to algorithmic opacity, reduced autonomy, and perceptions of technological overreach may also emerge. These potentially ambivalent effects imply that AI could interact with green talent management in complex ways, either supporting or constraining its ability to shape employees’ attitudes and identities. Overall, we explore how AI influences civil servants’ green organizational identity and satisfaction in the context of green talent management. By incorporating this novel influencing factor, this study contributes to the literature on the integration of talent management and AI, and enriches the discussion on AI in the field of public management.

## 2 Literature review and hypothesis development

### 2.1 Green talent management

Green talent management refers to the strategic allocation and cultivation of talent within an organization that possesses deep environmental awareness, sustainability capabilities, and specialized green skills (Ogbeibu et al., 2022). These individuals not only have the technical expertise and knowledge but also the ability to integrate environmental values into the organization’s core culture and management practices. They are committed to leveraging their capabilities and actions to help the organization achieve its environmental goals, thereby promoting green and sustainable development (Odugbesan et al., 2023; Song and Xie, 2019; Umair et al., 2023). For example, organizations can provide employees with training and development opportunities to enhance their environmental awareness, capabilities, and skills. They can also establish performance evaluation systems linked to environmental sustainability goals and incentivize employees to engage in sustainable development activities through reward and motivation mechanisms. Furthermore, fostering a green organizational culture, providing effective support resources, and encouraging employees to innovate in sustainability are essential aspects of green talent management (Elzek et al., 2023; Odugbesan et al., 2023).

Building on the strategic HRM and green HRM literature, scholars have increasingly argued that green talent management encompasses two fundamentally distinct managerial logics and, therefore, should be conceptualized as a dual-dimensional construct consisting of green hard talent management (GHTM) and green soft talent management (GSTM). This distinction is not merely semantic but reflects two theoretically grounded approaches to how organizations mobilize and integrate green talent. GHTM is a mechanistic, market-oriented talent management strategy that views green talent as crucial resources to be effectively and efficiently managed and controlled through strict performance evaluation systems, hierarchical organizational cultures, and bureaucratic work structures. In contrast, GSTM focuses more on the humanistic aspects of talent management. It emphasizes effective communication, talent involvement in decision-making processes, organizational support for talent well-being and health, and actively supports and commits to the development and retention of green talent by incentivizing talented team members to undertake well-defined ecological initiatives (Odugbesan et al., 2023; Ogbeibu et al., 2022; Umair et al., 2023). This two-dimensional division is theoretically grounded in the well-established distinction between hard and soft HRM practices, which reflect fundamentally different managerial logics.

Previous studies have explored the relationship between green talent management and employee retention (Ma et al., 2023), innovative work behavior (Odugbesan et al., 2023), and sustainable performance of enterprises (Umair et al., 2023), constructing various research models to investigate mediating and moderating mechanisms, as shown in Table 1. However, existing research has several limitations. First, the conclusions are ambiguous and sometimes contradictory. For example, Ogbeibu et al. (2022) found that green talent management, whether GHTM or GSTM, increased employees’ turnover intentions. However, Ma et al. (2023) indicated that green talent management positively impacts employee retention, suggesting it can reduce turnover intentions. The reasons for these discrepancies are complex and may be influenced by sample differences, highlighting the need for further research to examine the effects of green talent management. Second, most existing studies are conducted in for-profit organizations, leading to a lack of evidence on the implementation effects of green talent management in public organizations like government department. Although Odugbesan et al. (2023) focused on higher education institutions, which can be non-profit or for-profit, there is still a lack of attention to civil servants, a specific group typically with high social status, job security, and clear career advancement paths. It might result in differences in the implementation of green talent management compared to general corporate employees. Third, the models proposed in existing studies need further refinement. Some studies lack exploration of mediating mechanisms, such as those by Ogbeibu et al. (2022) and Odugbesan et al. (2023). Although Ma et al. (2023) addressed mediating mechanisms, their unidimensional approach hinders a comprehensive understanding of the differentiated impacts of the two green talent management strategies.

**Table 1:** Literature review of green talent management.

Authors	Research content	Subjects	Country	Research model
Ogbeibu et al. (2022)	Their study explored the relationship between green talent management and turnover intention.	Employees of 49 different manufacturing organizations (for-profit organization)	Nigeria	Independent Variables: green hard talent management and green soft talent management. Dependent Variable: turnover intention. Moderating Variables: leader STARA competence and digital task interdependence.
Ma et al. (2023)	Their study explored the relationship between green talent management and employee retention.	Service employees in the tourism firms (for-profit organization)	Pakistan	Independent Variable: green talent management (single dimension). Dependent Variable: employee retention. Mediating Variable: green organizational identity Moderating Variable: green shared vision.
Odugbesan et al. (2023)	Their study explored the relationship between green talent management and innovative work behavior.	Academic staff in the higher education institutions (for-profit organization or nonprofit organizations)	Northern Cyprus	Independent Variables: green hard talent management and green soft talent management. Dependent Variable: innovative work behavior. Moderating Variables: transformational leadership and artificial intelligence.
Umair et al. (2023)	Their study explored how green talent management and environmental corporate social responsibility influence the sustainable performance of enterprises.	Employees of the selected commercial banks (for-profit organization)	Oman	Independent Variables: environmental CSR, green hard talent management, green soft talent management, innovative work behavior and employees’ green performance (Structural Equation Modeling). Dependent Variable: sustainable performance Moderating Variable: green transformational leadership
The current research	The current study focuses on the implementation effects of green talent management in government agencies, specifically how it influences the satisfaction of civil servants.	347 civil servants (non-profit organizations)	China	Independent Variables: green hard talent management, green soft talent management. Dependent Variable: job satisfaction. Mediating Variable: green organizational identity. Moderating Variable: artificial intelligence

In summary, the current study investigates the impact of green talent management on civil servants’ job satisfaction. By constructing a comprehensive research model that includes mediating and moderating mechanisms and examining green talent management in two dimensions, this study contributes to the existing literature. In the next subsection, we will propose our research hypotheses based on previous studies.

### 2.2 Green talent management and job satisfaction

Green talent management emphasizes the establishment of formal environmental protection procedures and policies within talent management. By setting environmental goals and regulations and incorporating them into employee performance evaluations and incentive mechanisms, organizations encourage employees to take responsibility for the environment (Azhar and Yang, 2022; Elzek et al., 2023). Under green talent management, the values of green and sustainable development are cultivated, and employees are provided with opportunities to engage in environmental actions, such as participating in environmental protection-related projects (Ogbeibu et al., 2022). Organizations foster an organizational culture that supports environmental protection and sustainable development, encouraging employees to practice green concepts in their daily work. Training is provided to enhance employees’ knowledge and skills in environmental management, energy efficiency, and resource conservation. Performance evaluations include environmental and sustainability indicators, and reward and recognition mechanisms are used to motivate employees to adopt green practices and innovations in their work (Ma et al., 2023; Odugbesan et al., 2023; Umair et al., 2023).

The GHTM is a mechanistic talent management strategy that promotes sustainability through strict performance evaluations and hierarchical organizational cultures. It aims to guide employees to engage in behaviors that align with environmental principles, such as energy conservation, waste reduction, and pollution prevention, while emphasizing the organization’s commitment to environmental sustainability through the implementation of environmental protection measures and policies in talent management (Ogbeibu et al., 2022; Umair et al., 2023). In contrast, GSTM is more human-centered, focusing on internal communication and employee well-being. This strategy encourages employees to actively participate in the organization’s environmental protection and sustainable development initiatives by enhancing their sense of connection to the organization (Odugbesan et al., 2023; Ogbeibu et al., 2022). GSTM fosters a positive work environment and employee engagement by providing work resources such as a good work environment, welfare benefits, and career development opportunities, thereby improving employees’ job satisfaction.

Given that GHTM and GSTM have been well conceptualized in prior research, it is reasonable to expect that these two strategies exert different effects on employees. Existing studies generally suggest that GSTM, with its emphasis on participation, communication, and support for well-being, is more likely to satisfy employees’ psychological needs and therefore enhance their job satisfaction; In contrast, GHTM is often viewed as potentially constraining, which may suppress employees’ sense of autonomy or increase work pressure, thereby reducing satisfaction (Odugbesan et al., 2023; Ogbeibu et al., 2022; Umair et al., 2023; Ma et al., 2023). Drawing on this established literature and conventional theoretical expectations, we propose that GSTM should positively influence civil servants’ job satisfaction, whereas GHTM may have a hindering effect. Based on the above reasoning, this study proposes the following hypotheses:

*H1*: Green soft talent management (GSTM) positively affects civil servants' job satisfaction.

*H2*: Green hard talent management (GHTM) negatively affects civil servants' job satisfaction.

### 2.3 The mediating role of green organizational identity

The concept of green organizational identity originates from organizational identity, representing a subset focused on environmental protection and sustainability (Joshi and McKendall, 2018; Ran and Golden, 2011). Green organizational identity refers to the unique image and sense of identification that an organization establishes among its members by integrating the principles of environmental protection and sustainable development into its culture, strategy, practices, and behaviors (Chen, 2011; Song et al., 2019). Green organizational identity involves not only the internal alignment of members with the organization’s environmental values and behaviors but also the external recognition and trust of the public regarding the organization’s environmental image (Kiani et al., 2024; Zhu et al., 2021). Although research in public administration has affirmed the importance of organizational identity, demonstrating its ability to enhance civil servants’ psychological well-being and happiness (Hameed et al., 2022), organizational citizenship behavior (Shim and Faerman, 2017), and job performance (Miao et al., 2019), the exploration of green organizational identity within public organizations remains limited.

This study posits that green talent management can influence civil servants’ job satisfaction through the mediating role of green organizational identity. Organizational Identification Theory posits that employees develop a sense of oneness with their organization when its values, goals, and practices resonate with their own self-concept (Ashforth and Mael, 1989; Dutton et al., 1994). Green talent management conveys strong pro-environmental values and signals organizational commitment to sustainability, which employees can internalize as part of their identity. When civil servants perceive that their organization integrates sustainability into its practices and values, they are more likely to experience stronger green organizational identity, which subsequently enhances job satisfaction.

Building on this theoretical foundation, green talent management can further instill in employees a sense of meaning and personal responsibility in pursuing outcomes that hold value for themselves or for society at large (Kravariti and Johnston, 2020). In the public sector context, it can help employees feel that the organization acknowledges their public service values and realizes them through their work. By doing so, it strengthens individual identification with the organization. This represents a positive internalization process, ultimately forming a bond between the employee and the organization, linking personal self-concept with organizational membership (Macfarlane et al., 2012; Mensah, 2019). This connection can be achieved both cognitively and emotionally. Under the influence of green talent management, employees feel part of the organization, internalize its values, and take pride in and identify with their role. Additionally, as green talent management emphasizes social responsibility aspects such as environmental protection and sustainable development, it closely aligns with the public service motivation of civil servants (Miao et al., 2019). This philosophy emphasizes altruism, moral responsibility, and a greater focus on public interests, making it highly compatible with the professional identity of civil servants. Therefore, when the organization’s management philosophy aligns with their values, they feel satisfied and enjoy their identity as members of the organization. The study by Ma et al. (2023) also indicates that green talent management can enhance members’ green organizational identity. Based on the above analysis, we propose the following hypothesis:

*H3*: Green organizational identity mediates the relationship between green talent management and civil servants’ job satisfaction.

### 2.4 The moderating role of artificial intelligence

The application of artificial intelligence (AI) in the public sector is becoming increasingly widespread, aiming to design, build, adopt, and evaluate algorithms and computational technologies to enhance public administration and policy-making (Li et al., 2023; Neumann et al., 2024). At the core of AI, algorithms serve as fundamental components that help the public sector achieve efficient, low-cost, or “neutral” solutions (Wirtz and Müller, 2019). These algorithms have been applied in various fields, such as transportation (Kouziokas, 2017), criminal justice (Whitford et al., 2020), and policing (Meijer et al., 2021).

As AI is progressively deployed in public sectors like government, its impact has attracted significant scholarly interest. Li et al. (2023) argue that AI can provide substantial value to public administration departments. The first type is operational public value, which means that AI effectively addresses the challenges of digital government transformation, reflected in the reduction of administrative burdens and the technological difficulties of information sharing and collaboration. The second type is strategic public value, meaning that AI can transform public management services from isolated operations to cross-departmental collaborative interactions, thereby improving the quality of public services and transforming the orientation of government services. Other studies have discussed specific issues of AI application in public organizations, such as the alignment of AI integration in public organizations (Selten et al., 2023; Wirtz and Müller, 2019), public attitudes toward the government’s adoption of AI (Wang et al., 2023), and the administrative risks and governance issues related to AI use (Bullock, 2019).

The application of AI may also have an additional impact on talent management, as AI can assist management in formulating and adjusting sustainable development strategies, ensuring alignment with the long-term goals of the organization (Roppelt et al., 2024; Rožman et al., 2022). Using algorithms and big data, AI can enhance talent training, goal setting, and performance evaluation. For instance, AI can analyze employees’ learning styles and needs to create personalized training programs and use Virtual Reality (i.e., VR) and Augmented Reality (i.e., AR) resources to enhance civil servants’ green skills and knowledge (Kozinets, 2023). By analyzing and learning from historical data, organizations can set more suitable interim goals for employees, improve the accuracy and fairness of evaluations, and foster a sense of identification with the organization (Wirtz and Müller, 2019).

However, AI’s role in management is not uniformly beneficial. Instead, it exhibits a selective amplification effect, meaning that its impact depends on the specific logic embedded within the management practice. While prior research highlights AI’s advantages, scholars increasingly note that AI systems may also intensify organizational control, bureaucratic rigidity, and psychological pressure (Kellogg et al., 2020). Algorithmic systems heighten visibility and traceability, reduce managerial discretion, and enforce rule compliance through quantification and real-time monitoring. These features may undermine employees’ autonomy and foster feelings of depersonalization, thereby reducing identification with the organization (Kellogg et al., 2020). Similarly, technostress research suggests that data-driven evaluation systems can increase perceived surveillance, workload, and strain (Ayyagari et al., 2011). Taken together, these perspectives imply that AI does not exert a uniform influence across talent management systems; rather, it tends to magnify the dominant characteristics already inherent in each management strategy. Building on this view, we argue that AI is likely to strengthen the diverging effects of GHTM and GSTM.

GHTM emphasizes standardization, formalized procedures, and performance discipline. When combined with AI’s algorithmic monitoring and datafication mechanisms, these characteristics become more salient and constraining. AI-driven evaluation systems may reduce ambiguity but simultaneously diminish flexibility and interpersonal discretion, making performance requirements more rigid and amplifying feelings of depersonalization and organizational distance. As algorithmic systems enforce strict compliance and intensify bureaucratic controls, employees may experience lower autonomy and heightened alienation, thereby weaken green organizational identity and further reduce job satisfaction. Therefore, AI is expected to strengthen the negative pathway from GHTM to job satisfaction via GOI. Thus, we propose the following hypotheses:

*H4*: Artificial intelligence (AI) moderates the relationship between green hard talent management (GHTM) and green organizational identity (GOI). Specifically, through the mediating role of green organizational identity, AI strengthens the negative impact of GHTM on job satisfaction.

In contrast, GSTM emphasizes support, communication, developmental opportunities, and value alignment. AI can selectively amplify these soft practices by enabling individualized feedback, adaptive learning support, real-time communication, and personalized developmental recommendations. Through intelligent assistance tools and data-driven coaching, employees may perceive greater organizational care, enhanced capability development, and clearer alignment with green values and goals. These mechanisms strengthen GOI and ultimately increase job satisfaction. Therefore, in the context of GSTM, AI acts as a positive catalyst that reinforces the beneficial effects of soft talent management on employee outcomes. These arguments suggest that AI does not exert a uniform influence but instead produces differentiated reinforcement effects depending on whether the underlying talent management approach is hard or soft in nature, and the following hypotheses was proposed:

*H5*: Artificial intelligence (AI) moderates the relationship between green soft talent management (GSTM) and green organizational identity (GOI). Specifically, through the mediating role of green organizational identity, AI strengthens the positive impact of GSTM on job satisfaction.

**Figure 1:**
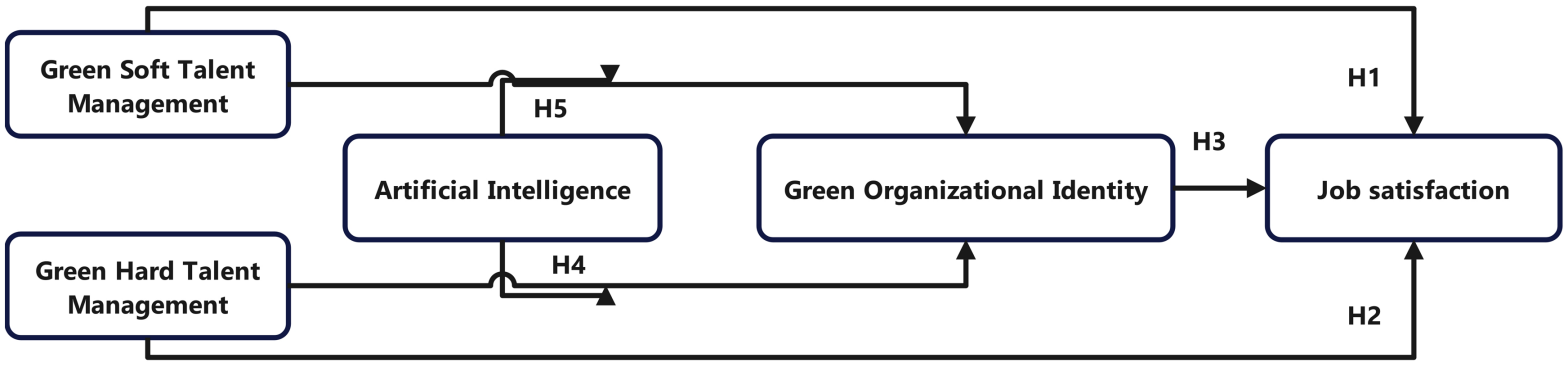
Moderating effects exerted by AI between GHTM and GOI.

In summary, our research model is illustrated in Figure 1.

## 3 Methods

This study employed an anonymous online questionnaire and did not involve any interventions or the collection of sensitive personal data. In accordance with the institutional policies of Lanzhou University of Finance and Economics, ethical review and approval were not required for this type of research. All procedures were conducted in compliance with relevant guidelines and regulations. Informed consent was obtained from all participants prior to data collection.

### 3.1 Samples and research design

We used a purposive sampling strategy to recruit respondents who met the eligibility criteria for civil servants in China. Participants were recruited through Credamo, a widely used professional survey platform, and only individuals who self-identified as current civil servants and passed attention checks were included in the final dataset. Although Credamo does not provide full coverage of all civil servant groups, it offers access to a diverse pool of administrative personnel across various regions and government departments (Chen et al., 2023; Fu et al., 2020; Miao et al., 2024; Wang and Zhang, 2024). Data collection lasted for 2 months, during which we received 430 questionnaires from government employees across 28 provinces. Subsequently, we meticulously checked all the questionnaires and conducted data cleaning. First, we removed low-quality responses based on completion time, deleting those with times significantly below the average. Attention check items were included in the survey (a simple arithmetic question and a question requiring a specified response), and responses failing these checks were deemed invalid. Finally, we eliminated 83 questionnaires due to inconsistent responses. After screening, we obtained 347 valid questionnaires for data analysis, resulting in an effective rate of 80.70%. Detailed sample information is provided in Table 2.

**Table 2:** Sample descriptive statistical information.

Categories	Items	Sample size	Percentage (%)
Genders	Male	116	33.43
	Female	231	66.57
Age	18–24 years old	38	10.95
	25–31 years old	133	38.33
	32–38 years old	136	39.19
	39–45 years old	13	3.75
	46–52 years old	22	6.34
	53 years and over	5	1.44
Working experience	Less than 1 year	24	6.92
	1 to 5 years	140	40.35
	5 to 10 years	121	34.87
	More than 10 years	62	17.87
Educational background	High school and below	3	0.86
	Associate’s Degree	21	6.05
	Bachelor’s Degree	263	75.79
	Master’s Degree	51	14.70
	PhD or higher	9	2.59
Positions	Managerial and positions	196	56.48
	Non-managerial positions	151	43.52
Monthly income	Less than 3,000 yuan	19	5.48
	3,000–6,000 yuan	90	25.94
	6,000–9,000 yuan	150	43.23
	9,000–12,000 yuan	50	14.41
	12,000–15,000 yuan	16	4.61
	More than 15,000 yuan	22	6.34

*N* = 347.

### 3.2 Measurement

Core variables were measured using a 5-point Likert scale (1 = strongly disagree, 5 = strongly agree). All scales were presented to participants in Chinese. As the original scales were developed in English, we employed the commonly used translation and back-translation procedures to ensure the accuracy of the Chinese version. The scales used in this study were adapted from previous research and adjusted to fit the Chinese context. Gender, age, years of work experience, education, position, and income were used as control variables.

We measured green soft talent management (GSTM) and green hard talent management (GHTM) based on the study by Ogbeibu et al. (2022). GSTM included six items, one example being: “My organization cares about my well-being and offers considerable support for my welfare when executing green-centered initiatives.” GHTM included four items, one example being: “Environmental sustainability initiatives in my organization are driven by a high level of bureaucracy.” Green organizational identity (GOI) was measured using four items adapted from Chen (2011), one example being: “I have a strong sense of the organization’s history about environmental management and protection.” The measurement of artificial intelligence (AI) was based on the study by Odugbesan et al. (2023) and included four items, one example being: “Artificial intelligence can help me in making important decisions in the organization.” Job satisfaction is measured using a single question item: “Overall, I am satisfied with my job” (Fisher et al., 2016). It should be noted that the use of a single-item measure for overall job satisfaction is well established in organizational research, with meta-analytic and validation evidence showing that such global single-item assessments demonstrate adequate validity and correlate strongly with multi-item scales (Dolbier et al., 2005; Nagy, 2002; Wanous et al., 1997).

## 4 Data analysis and results

The SPSS is a widely-used tool in academic data analysis due to its ease of use, comprehensive functionality, flexibility, and reliability. This study utilized SPSS 25.0 for data analysis. We first conducted reliability and validity tests, employing the widely accepted factor analysis methods for relevant indices, followed by linear regression and variance analysis to test our research model.

### 4.1 Reliability and validity testing

First, common method bias (CMB) is an important aspect affecting research reliability. We reduced the impact of CMB in two ways: one, by conducting anonymous surveys and collecting data from multiple sources (questionnaires from 28 different provinces in China) to reduce bias and enhance the generalizability of the results; two, by incorporating specific designs in the questionnaire, such as reverse-coded items and attention check items, to mitigate the severity of CMB. Harman’s single-factor test is commonly used to assess the severity of CMB. The KMO and Bartlett’s test of sphericity results indicated that KMO = 0.944 > 0.9, *χ*^2^ = 3656.217 (*p* < 0.001), meeting the conditions for factor analysis. The factor analysis results showed that the first factor explained 47.619% of the variance (below the critical value of 50%), suggesting that CMB has a minimal impact on the validity of the study.

Second, common reliability and validity tests are performed using indicators such as internal consistency coefficient (Cronbach’s Alpha), average variance extracted (AVE), and composite reliability (CR). The test results are shown in Table 3.

**Table 3:** Results of reliability and convergent validity tests.

Variables	Items	Loadings	CR	AVE	Cronbach’s alpha
Green Hard Talent Management	GHTM1	0.827	0.8357	0.5622	0.714
	GHTM2	0.804			
	GHTM3	0.707			
	GHTM4	0.647			
Green Soft Talent Management	GSTM1	0.761	0.9058	0.6161	0.912
	GSTM2	0.764			
	GSTM3	0.762			
	GSTM4	0.779			
	GSTM5	0.819			
	GSTM6	0.822			
Green Organizational Identity	GOI1	0.767	0.8277	0.5461	0.863
	GOI2	0.775			
	GOI3	0.713			
	GOI4	0.698			
Artificial Intelligence	AI1	0.797	0.7864	0.4878	0.702
	AI2	0.615			
	AI3	0.822			
	AI4	0.512			

Third, we conducted confirmatory factor analysis (CFA) to test model fit. The four-factor model (i.e., GHTM, GSTM, GOI, and AI) demonstrated acceptable model fit (*χ*^2^/df = 3.48, RMESA = 0.08, IFI = 0.91, CFI = 0.91, TLI = 0.90), which was superior to alternative models (see Table 4). Additionally, Table 5 presents the correlation analysis results of all variables.

**Table 4:** Results of model fitting.

Models	*χ* ^2^	*χ*^2^/df	RMESA	IFI	CFI	TLI
Four-factor model	449.54	3.48	0.08	0.91	0.91	0.90
Three-factor model	456.19	3.46	0.08	0.91	0.89	0.89
Two-factor model	463.29	3.46	0.08	0.90	0.89	0.89
single-factor model	567.43	4.20	0.10	0.88	0.87	0.86

Four-factor model: GHTM, GSTM, GOI, AI; three-factor model: GHTM + GSTM, GOI, AI; two-factor model: GHTM + GSTM + GOI, AI; single-factor model: GHTM + GSTM + GOI + AI.

**Table 5:** Results of Pearson correlation analysis.

Variables	Mean	SD	1	2	3	4
1 GHTM	3.72	0.80	–			
2 GSTM	3.76	0.89	0.69**	–		
3 GOI	3.90	0.87	0.70**	0.86**	–	
4 AI	3.65	0.71	0.31**	0.48**	0.46**	–
5 JS	5.72	1.08	0.42**	0.61**	0.62**	0.42*

*p < 0.05, **p < 0.01, ***p < 0.001.

### 4.2 Hypothesis testing

We used SPSS 25.0 to test our hypotheses. Models 1 and 3 were used to verify the main effects, while Models 2 and 4 were employed to examine the mediation effects. As shown in Table 6, there is a significant positive impact of GSTM on job satisfaction (Model 1, *B* = 0.57, *p* < 0.001), and GHTM also shows a significant positive effect on job satisfaction (Model 3, B = 0.37, *p* < 0.001). Therefore, Hypothesis 1 is supported, but Hypothesis 2 is not. Specifically, the relationship between GHTM and job satisfaction is contrary to our expectations, a potential reason for which will be discussed in the discussion section.

**Table 6:** Test results for main and mediating effects.

Variables	Model 1	Model 2	Model 3	Model 4
Gender	−0.02	−0.00	−0.07	0.00
Age	0.19^**^	0.17^**^	0.18^*^	0.16^*^
Working experience	−0.09	−0.10	−0.03	−0.11
Educational background	0.09^*^	0.11^*^	0.04	0.12^*^
Positions	−0.10^*^	−0.09^*^	−0.11^*^	−0.09^*^
Monthly income	0.05	0.05	0.10^*^	0.06
GHTM			0.37^***^	−0.07
GSTM	0.57^***^	0.28^***^		
GOI		0.35^***^		0.64^***^
ΔR^2^	0.42	0.03	0.25	0.19

*p < 0.05, **p < 0.01, ***p < 0.001.

According to the results of Model 2, after adding GOI into the regression between GSTM and job satisfaction, the mediation effect is significant (*B* = 0.35, *p* < 0.001), and GSTM remains significant (*B* = 0.28, *p* < 0.001), indicating that GOI partially mediates the relationship between GSTM and job satisfaction. According to the results of Model 4, after adding GOI into the regression between GHTM and job satisfaction, the mediation effect is significant (*B* = 0.64, *p* < 0.001), but GHTM is no longer significant (*B* = −0.07, *p* > 0.05), indicating that GOI fully mediates the relationship between GHTM and job satisfaction. Therefore, Hypothesis 3 is supported by the data. The discussion on partial and full mediation will be elaborated in the discussion section.

**Figure 2:**
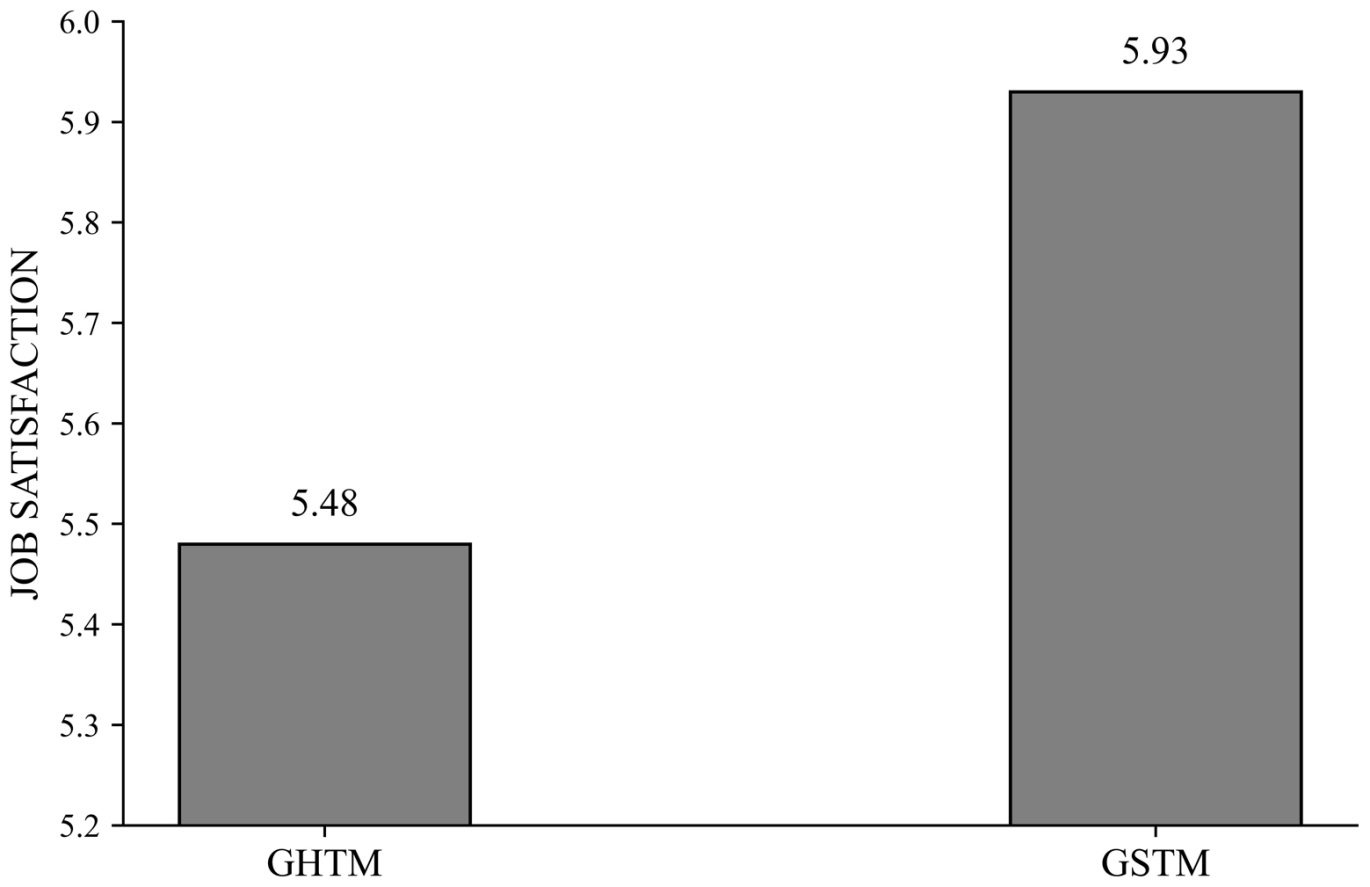
Research model.

Given the above results, which show that both GHTM and GSTM have significant positive impacts on job satisfaction, we further explored the intensity of these impacts using analysis of variance (ANOVA). We calculated the average scores for the items corresponding to GHTM and GSTM, subtracted one from the other, and computed the median. Participants with scores above the median were classified into the GHTM group, and those below into the GSTM group. A one-way ANOVA showed significant differences in job satisfaction between groups [*M*_GHTM_ = 5.48, *M*_GSTM_ = 5.93, *F*_(1,345)_ = 15.512, *p* < 0.001], as illustrated in Figure 2. This indicates that the GSTM group has significantly higher job satisfaction than the GHTM group, suggesting that the GSTM strategy is more effective in enhancing job satisfaction.

Subsequently, we tested the moderating effects. By centering the independent and moderating variables and constructing interaction terms, we incorporated them into the regression models, generating four models as shown in Table 7. Models 5 and 6 were used to examine the moderating effect of AI on the GHTM-GOI pathway, while Models 7 and 8 were used to test the moderating effect of AI on the GSTM-GOI pathway. As indicated by the results of Model 6, even after adding the interaction term, the positive effect of GHTM on GOI remained significant (*B* = 0.58, *p* < 0.001), and the interaction term was also significant (*B* = −0.11, *p* < 0.01). This suggests that AI moderates the effect of GHTM on GOI by weakening its positive influence. However, due to the inconsistency in the direction of the effect, Hypothesis 4 was only partially supported. The results of Model 8 indicated that the moderating effect of AI on the GSTM-GOI relationship was not statistically significant. Therefore, Hypothesis 5 was not supported.

**Table 7:** Test results for moderating effect.

Variables	Model 5	Model 6	Model 7	Model 8
Gender	−0.10^*^	−0.07	−0.03	−0.02
Age	0.03	0.06	0.07	0.07^*^
Working experience	0.12^*^	0.07	0.04	0.04
Educational background	−0.12^*^	−0.10^*^	−0.04	−0.04
Positions	−0.04	−0.01	−0.03	−0.02
Monthly income	0.06	0.10^*^	−0.01	0.01
GHTM	0.67	0.58		
GSTM			0.83	0.81
AI		0.25		0.05
GHTM × AI		−0.11^*^		
GSTM × AI				−0.01
ΔR^2^	0.55	0.06	0.76	0.00

*p < 0.05.

**Figure 3:**
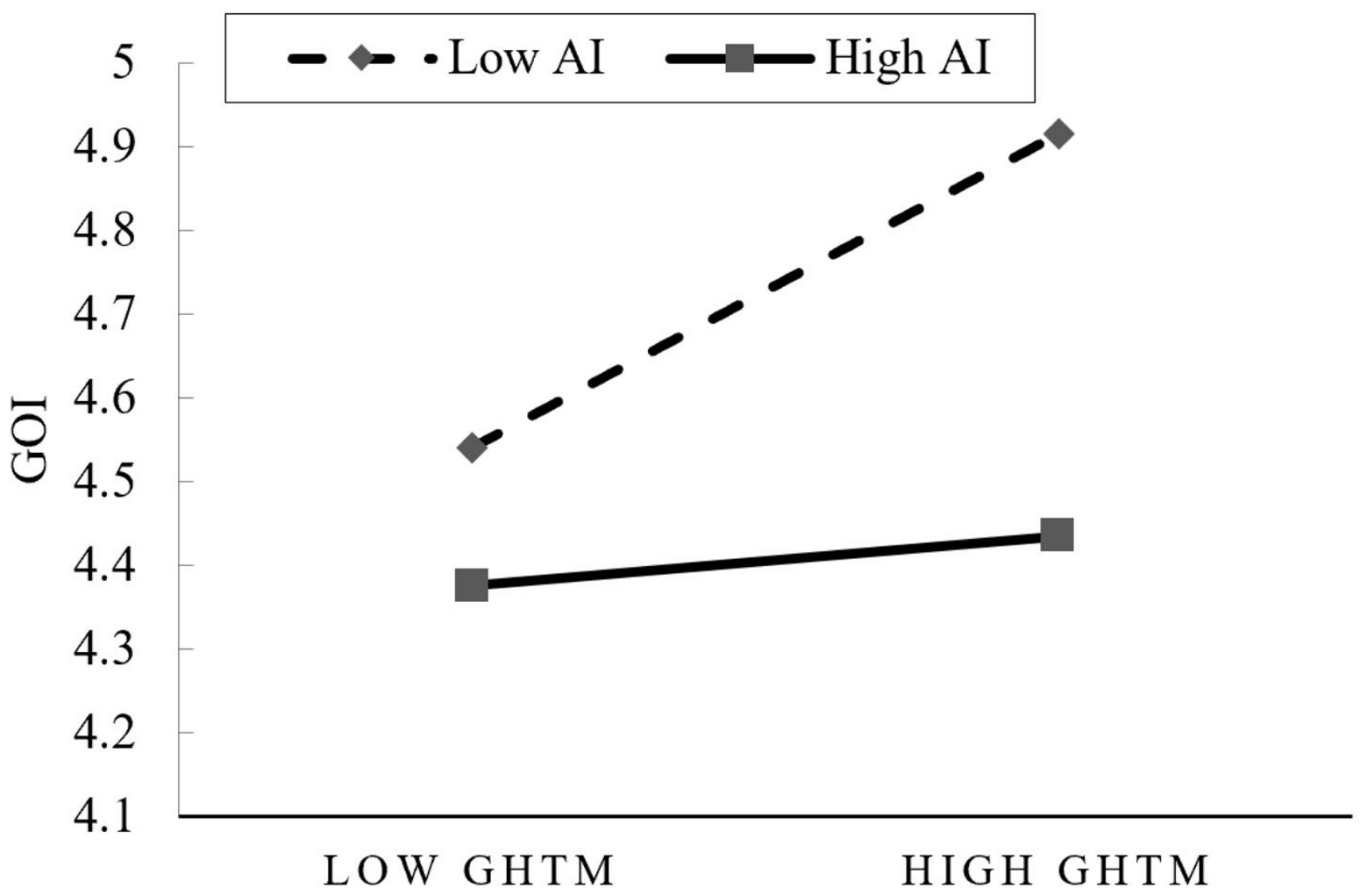
Difference in the effect of GHTM and GSTM on job satisfaction.

Finally, to more intuitively illustrate the moderating effect of AI on the GHTM-GOI relationship, we divided GHTM and AI into high and low levels (based on the mean plus or minus one standard deviation) and created a simple slope plot, as shown in Figure 3. In the high AI context, the positive effect of GHTM on GOI was weaker, as evidenced by the flatter slope of the line in the figure. This indicates that AI hinders the enhancement of employees’ GOI through GHTM, thereby negatively impacting the improvement of job satisfaction.

## 5 Discussion

Given the critical role that government and public organizations play in maintaining public interest and promoting social welfare, fostering and retaining talent in the public administration sector to enhance sustainable development has become a focal point in recent research (Blijleven and Van Hulst, 2021; Chen et al., 2023; Kravariti and Johnston, 2020). The primary contribution of this study lies in the theorization and evaluation of a previously unexplored moderated mediation model. This model incorporates two dimensions of green talent management (GHTM and GSTM) along with green organizational identity and artificial intelligence to jointly examine civil servant satisfaction. Based on data from 347 survey responses from China, we tested our research model and derived several meaningful insights.

### 5.1 Both forms of green talent management enhance job satisfaction

This study first reveals that both GHTM and GSTM significantly enhance civil servants’ job satisfaction. The positive effect of GSTM aligns well with prior research (Odugbesan et al., 2023; Umair et al., 2023), suggesting that soft, human-centered management practices (e.g., communication, support, and attention to employee well-being) effectively strengthen civil servants’ engagement and psychological fulfillment. However, contrary to our initial hypothesis (H2), GHTM also demonstrated a positive impact on job satisfaction, a finding that warrants deeper examination.

Previous literature has often portrayed GHTM as a mechanical, institutionalized, and control-oriented management approach that relies on strict performance metrics, bureaucratic procedures, and structured task requirements to advance green goals. Such characteristics were assumed to generate pressure and negative emotions (Odugbesan et al., 2023; Umair et al., 2023). Yet, the civil servants examined in this study operate in a highly institutionalized environment defined by stability, clear role boundaries, and well-established career advancement systems. They generally show strong adaptation to bureaucratic structures, which may lead them to respond to GHTM quite differently from employees in corporate settings.

In the public sector, structured performance management and bureaucratic organizational arrangements are frequently regarded as safeguards of fairness, transparency, and procedural justice (Boyne, 2002; Pollitt, 2006). For civil servants, these practices do not necessarily impose additional burdens; instead, they provide clearer task expectations, more predictable career trajectories, and a stronger sense of organizational order. This institutional stability and predictability help reduce role ambiguity and uncertainty, ultimately fostering more positive work attitudes. As a result, civil servants tend to view GHTM as a management approach that ensures fair competition, maintains organizational justice, and offers well-defined promotion opportunities (Micacchi et al., 2023; Moynihan and Pandey, 2007; Wright, 2001).

This finding constitutes an important contribution of the present study, indicating that prior interpretations of GHTM may have been overly general. The effects of GHTM are not inherently negative but instead depend on the specific characteristics of the population being managed. For civil servants, who typically place high value on structure, fairness, and institutional clarity, GHTM can function as a constructive management approach that genuinely enhances their work experiences.

### 5.2 Green organizational identity mediates both forms of green talent management

Our findings confirm that green organizational identity plays a significant mediating role in the relationship between green talent management and civil servants’ job satisfaction, thereby providing strong support for H3. Green organizational identity reflects employees’ perceptions of their organization’s environmental values, commitment, and reputation in sustainability, and prior studies have emphasized its importance as a psychological mechanism linking green management practices to employee outcomes (Chen, 2011; Ma et al., 2023). Consistent with this view, our findings show that green organizational identity fully mediates the relationship between GHTM and job satisfaction. In other words, GHTM enhances civil servants’ job satisfaction primarily by strengthening their identification with the organization’s green mission and values.

For the GSTM pathway, however, green organizational identity functions as a partial mediator. This indicates that while GSTM promotes job satisfaction partly by elevating employees’ identification with the organization’s environmental commitments, additional psychological or behavioral mechanisms may also contribute to its positive effects. Future studies could further investigate alternative mediators, such as perceived organizational support, psychological empowerment, or meaningfulness of work.

Overall, these findings extend the work of Ma et al. (2023), who demonstrated the mediating role of green organizational identity in the relationship between green talent management and employee retention. By separately examining the two dimensions of green talent management, our study refines and enriches the theoretical understanding of how different green management strategies shape employee attitudes through distinct psychological pathways.

### 5.3 AI weakens the positive effects of GHTM among civil servants

Building on the earlier findings, our results show that both GHTM and GSTM are positively associated with civil servants’ job satisfaction, despite our original expectation that GHTM would exert a negative effect. This reversal of the H2 assumption has direct implications for the evaluation of our moderation hypotheses. H4 and H5 were formulated on the premise that GHTM is potentially harmful while GSTM is beneficial, and that AI would amplify these diverging effects by strengthening the negative pathway of GHTM and the positive pathway of GSTM. Given that GHTM turned out to be beneficial rather than detrimental, this underlying theoretical premise no longer holds, and both H4 and H5 must therefore be regarded as empirically rejected.

However, the analysis still revealed a statistically significant interaction between AI and GHTM in predicting green organizational identity, and consequently job satisfaction. Importantly, this interaction operates in the opposite direction to what we initially hypothesized: higher levels of AI usage weaken, rather than strengthen, the positive effect of GHTM on green organizational identity. In other words, in a civil service context where GHTM by itself enhances job satisfaction because bureaucratic, structured, and performance-oriented systems are viewed as fair and predictable, the introduction of AI into these hard management practices may reduce their otherwise positive effects on employees’ identity formation.

This unexpected pattern can be better understood through the lenses of technological alienation and techno-skepticism. From a technological alienation perspective, AI-enabled GHTM often relies on algorithmic performance evaluations, automated monitoring, and data-driven optimization of work processes (Kellogg et al., 2020; Tambe et al., 2019). While such systems may improve efficiency and precision, they can also make civil servants feel that their professional judgment, experiential knowledge, and individual contributions are being displaced or overshadowed by technology. As a result, the same structured and bureaucratic arrangements that previously signaled fairness and order may start to feel depersonalized and overly mechanistic when mediated by AI, thereby weakening employees’ sense of involvement and their identification with the organization’s green mission (Aroles et al., 2019). Techno-skepticism offers a complementary explanation. AI systems used in talent management and green performance monitoring are often complex and opaque, making it difficult for employees to fully understand how decisions are made, how data are processed, or on what basis evaluations are reached (Chuan et al., 2024; Schiff et al., 2022; Zhou et al., 2023). If civil servants question the transparency, fairness, or accountability of AI-supported decisions, they may begin to distrust the broader talent management strategy in which these systems are embedded. This skepticism can spill over to their perceptions of the organization’s green initiatives, leading them to distance themselves from the green organizational identity that GHTM is intended to foster.

These findings suggest that, in the civil service context, GHTM on its own can enhance job satisfaction by providing structure, predictability, and perceived fairness, but the introduction of AI into these hard management practices may erode part of this positive effect by generating feelings of alienation and skepticism. Rather than acting as a universal amplifier of green talent management, AI appears to function as a conditional dampening factor in the GHTM pathway. This not only clarifies why our original moderation hypotheses were not supported, but also offers a novel and theoretically meaningful insight into how AI can inadvertently undermine the positive impact of otherwise effective management practices (Bullock, 2019; Li et al., 2023; Taeihagh, 2021).

## 6 Limitations and future research

The limitations of this study require further explanation. First of all, as our data are cross-sectional and derived from China’s public sector, a cautious approach should be taken when generalizing the findings. Although Credamo is a widely used and reliable data collection platform in China, its user base consists primarily of digitally active participants, which may not fully capture the demographic and occupational characteristics of the broader civil servant population. Some civil servants—especially those in lower administrative levels, older age groups, or less digitally engaged positions—may be underrepresented on such platforms, potentially introducing sampling bias. While using Chinese data supplements existing research evidence, future studies need to expand the scope of surveys. For instance, all current research on green talent management has been conducted in developing countries, and subsequent scholars could focus on the implementation effects of green talent management in developed countries, employing multi-time-point and more rigorous survey methods.

Second, the mediating and moderating mechanisms through which green talent management operates could be further explored in future research, as our study only provided one possible pathway. In fact, green organizational identity exhibited full mediation in one pathway and partial mediation in another, suggesting the existence of other mediating pathways. Additionally, the role of AI in talent management warrants further investigation, as our study only conducted a preliminary exploration, leaving room for further refinement.

Finally, although we adopted a validated single-item measure of overall job satisfaction and prior meta-analytic studies have demonstrated its adequacy as a global indicator, this approach nonetheless presents certain limitations. Single-item measures do not allow for internal consistency reliability testing and may not fully capture the multidimensional nature of job satisfaction. Therefore, future research could employ multi-item or multidimensional job satisfaction scales to more comprehensively validate and extend our conclusions, ensuring greater precision in measuring employee attitudes across different organizational contexts.

## 7 Conclusion

This study shows that both green hard talent management (GHTM) and green soft talent management (GSTM) enhance civil servants’ job satisfaction, highlighting the distinctive suitability of these practices in the public sector. Green organizational identity emerged as a key mechanism, fully mediating the effect of GHTM and partially mediating the effect of GSTM. Although the proposed moderating hypotheses were not supported, the findings reveal an important and unexpected pattern: artificial intelligence weakens, rather than strengthens, the positive influence of GHTM on green organizational identity and, in turn, job satisfaction. These results refine understanding of how different forms of green talent management operate in government settings and provide a basis for future research on the boundaries and conditions under which AI supports—or potentially undermines—public-sector human resource practices.

## Data Availability

The raw data supporting the conclusions of this article will be made available by the authors, without undue reservation.
